# The Evaluation of More Lymph Nodes in Colon Cancer Is Associated with Improved Survival in Patients of All Ages

**DOI:** 10.1371/journal.pone.0155608

**Published:** 2016-05-19

**Authors:** Wouter B. aan de Stegge, Barbara L. van Leeuwen, Marloes A. G. Elferink, Geertruida H. de Bock

**Affiliations:** 1 Department of Surgery, Hospital Group Twente, Almelo / Hengelo, the Netherlands; 2 Department of Surgery, University Medical Centre Groningen, University of Groningen, Groningen, the Netherlands; 3 Netherlands Comprehensive Cancer Organisation, Utrecht, the Netherlands; 4 Department of Epidemiology, University Medical Centre Groningen, University of Groningen, Groningen, the Netherlands; INRS, CANADA

## Abstract

**Background:**

Improvement in survival of patients with colon cancer is reduced in elderly patients compared to younger patients. The aim of this study was to investigate whether the removal of ≥ 12 lymph nodes can explain differences in survival rates between elderly and younger patients diagnosed with colon cancer.

**Methods:**

In a population-based cohort study, all patients (N = 41,074) diagnosed with colon cancer stage I to III from 2003 through 2010 from the Netherlands Cancer Registry were included. Age groups were defined as < 66, 66–75 and > 75 years of age. Main outcome measures were overall and relative survival, the latter as a proxy for disease specific survival.

**Results:**

Over an eight years time period there was a 41.2% increase in patients with ≥ 12 lymph nodes removed, whereas the percentage of patients with the presence of lymph node metastases remained stable (35.7% to 37.5%). After adjustment for patient and tumour characteristics and adjuvant chemotherapy, it was found that for patients in which ≥ 12 lymph nodes were removed compared to patients with < 12 lymph nodes removed, there was a statistically significant higher overall survival (< 66: HR: 0.858 (95% CI, 0.789–0.933); 66–75: HR: 0.763 (95% CI, 0.714–0.814); > 75: HR: 0.734 (95% CI, 0.700–0.771)) and relative survival (< 66: RER: 0.783 (95% CI, 0.708–0.865); 66–75: RER: 0.672 (95% CI, 0.611–0.739); > 75: RER: 0.621 (95% CI, 0.567–0.681)) in all three age groups.

**Conclusions:**

The removal of ≥ 12 lymph nodes is associated with an improvement in both overall and relative survival in all patients. This association was stronger in the elderly patient. The biology of this association needs further clarification.

## Introduction

Colon cancer is one of the most common cancers in men and women [1]. Each year, worldwide, over one million cases are newly diagnosed and approximately 600 000 patients die of this disease. As the incidence increases with age, colon cancer occurs mainly in patients older than 65 years of age [2]. In the Netherlands, roughly 70% of patients are in this age group when diagnosed with colon cancer, with a peak incidence between 70–74 years [3]. Colon cancer survival has improved over the last few decades. However, this improvement in survival is reduced in elderly patients compared to younger patients [4,5]. For colon carcinomas the 5-year relative survival for patients of 75 years and older is 56%, compared to 60% in patients aged 65 to 74 years, and up to 65% in patients younger than 65 years [6].

The presence of nodal metastases at the time of surgical treatment has been found to be the most important determinant of prognosis in patients with localized colon cancer [7,8]. If there is nodal involvement in colon cancer, a patient is eligible for adjuvant chemotherapy [9]. Adequate staging of a tumour will lead to the just allocation of adjuvant therapy and improve survival [10,11]. Given the high local recurrence and mortality in stage II patients with lower number of removed lymph nodes, adjuvant chemotherapy is also appropriate for these patients [12]. Although these so called high-risk stage II and stage III patients are eligible for adjuvant treatment, especially in the elderly patients, the allocation depends on frailty and comorbidity. The number of removed lymph nodes depends among other things on the size of the resected specimen (surgeon dependent) and the accuracy of the pathologist. Due to several reasons, the removal of lymph nodes decreases with age [13].

The aim of this study was to investigate whether the removal of more (≥ 12) lymph nodes can explain differences in survival rates between elderly and younger patients diagnosed with colon cancer.

## Methods

Data were retrieved from the Netherlands Cancer Registry (NCR). The NCR has been collecting data on newly diagnosed cancers since 1989. The database contains data on over 95 percent of patients diagnosed with cancer in the Netherlands. The NCR uses the same code system as the World Health Organisation (WHO) and the International Association of Cancer Registries (IACR). Local pathological laboratories collect the pathological data. All these laboratories are affiliated to the Pathologische Anatomische Landelijke Geautomatiseerd Archief (PALGA), the nationwide Dutch network and registry of histopathology and cytopathology. The NCR is based on reports of all newly diagnosed malignancies in the Netherlands by PALGA. The NCR includes detailed information on patients’ characteristics, year of diagnosis, type of surgery, tumour characteristics (e.g. tumour location, tumour stage according to the UICC classification [14], morphology and differentiation grade), treatment, number of removed lymph nodes and number of lymph node metastases. Tumour location was defined as right-sided including transversum, left-sided and sigmoid. The sixth edition of TNM classification was used for staging the tumour. Patients’ vital status was obtained by linking the NCR to the municipality register. Follow-up was completed until December 2010. For patients who were still alive, this date was taken as censoring date; unless the patient emigrated, then the date of emigration was taken as censoring date.

**Fig 1:**
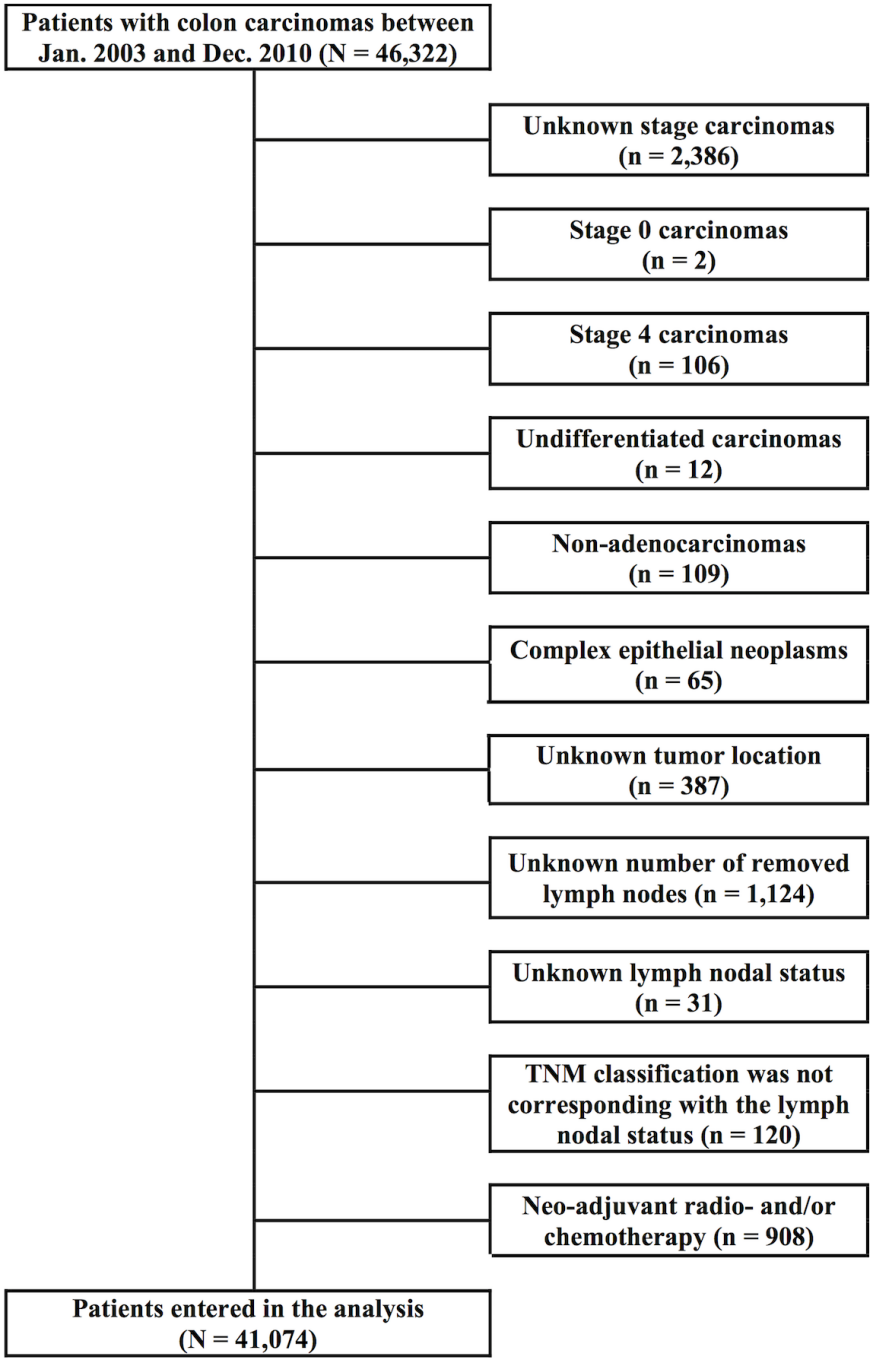
Flowchart of the in- and exclusion of patients with colon cancer in this analysis. Data were retrieved from the Netherlands Cancer Registry.

For this study, data from all patients diagnosed between January 2003 and December 2010 with adenocarcinomas of the colon, stage I-III, and differentiation grade I-III tumours (well—poorly differentiated), including those with an unknown differentiation grade, were retrieved (N = 46,322). Excluded were patients with an unknown tumour location (n = 387), unknown number of removed lymph nodes (n = 1,124), unknown lymph nodal status (n = 31) and patients for whom the TNM classification did not correspond with the lymph nodal status (n = 120). Patients who received neo-adjuvant treatment were also excluded (n = 908). After exclusion 41,074 patients were available for the analysis, see Fig 1.

No approval from a Medical Ethical Committee or Institutional board was needed for this study, since anonymized and de-identified data were retrieved from the Netherlands Cancer Registry.

Based on morphology, the tumours were divided into two groups. One group contained adenocarcinomas and a second group contained mucinous neoplasms. Based on clinical guidelines patients were also divided into two groups according to the number of removed lymph nodes: < 12 and ≥ 12. Three patient groups were established based on age at time of diagnosis: < 66 years, 66–75 years, and > 75 years. These age groups are frequently used in epidemiological studies in elderly patients with colon cancer.

### Statistics

Patients and clinical and pathological characteristics were presented, as well as changes over time in the percentage of patients with 12 of more lymph nodes removed per year of diagnosis and the percentage of patients with lymph node metastases per year of diagnosis stratified for the three age groups (< 66 years, 66–75 years, and > 75 years). Multivariate logistic regression analysis was performed to identify the variables that were associated with the removal of 12 or more lymph nodes.

Survival was estimated by the application of the Kaplan-Meier method for the patients in which 12 of more lymph nodes were removed versus those in which less than 12 lymph nodes were removed, stratified by age group and compared using log-rank tests. A multivariate Cox regression analysis was performed to analyse whether the removal of 12 or more lymph nodes for given age groups was associated with absolute survival. Therefore these variables were entered as interaction factors into the analysis. The analysis was adjusted for the covariates with trend-significant effects (*p <* 0.10) on the univariate analysis. All these analyses were carried out using SPSS (version 20.0; SPSS Inc, Chicago, III).

Relative survival, an estimation of disease-specific survival, was calculated as the ratio of the observed rates in cancer patients to expected rates in the general population using the Ederer method [15]. Relative excess risks (RER) of dying were estimated by means of multivariable relative survival analyses. STATA (version 14) was used for this analysis.

## Results

The median age at time of diagnosis was 72 years (IQR: 63–79; Table 1). Most patients had stage II cancer (44.7%) followed by 36.9% stage III cancer, and 18.4% stage I cancer.

**Table 1:** Patient, clinical and pathological characteristics (N = 41,074).

Characteristics	N (%)^a^
Age median (IQR)	72 (63–79)
< 66 years	12,584 (30.6)
66–75 years	13,088 (31.9)
> 75 years	15,402 (37.5)
Gender	
Male	20,817 (50.7)
Female	20,257 (49.3)
Tumour location	
Right (+ transversum)	20,112 (49.0)
Left	3,394 (8.2)
Sigmoid	17,568 (42.8)
Morphology	
Adenocarcinomas	34,244 (83.6)
Mucinous neoplasms	6,730 (16.4)
Differentiation grade	
Well differentiated	3,242 (7.9)
Moderately differentiated	28,088 (68.3)
Poorly differentiated	6,485 (15.8)
Unknown	3,259 (7.9)
Tumour invasion	
T1	2,441 (5.9)
T2	6,523 (15.9)
T3	26,854 (65.4)
T4	5,256 (12.8)
N stage	
N0	25,923 (63.1)
N1	10,188 (24.8)
N2	4,963 (12.1)
Stage	
Stage I	7,573 (18.4)
Stage II	18,350 (44.7)
Stage III	15,151 (36.9)
Year of diagnosis	
2003	4,301 (10.5)
2004	4,659 (11.3)
2005	4,899 (11.9)
2006	5,236 (12.7)
2007	5,367 (13.1)
2008	5,491 (13.4)
2009	5,655 (13.8)
2010	5,466 (13.3)
Adjuvant chemotherapy	
No	31,018 (75.5)
Yes	10,056 (24.5)
Follow-up months median (IQR)	39 (20–64)

^a^Unless specified otherwise

IQR Interquartile range

**Fig 2:**
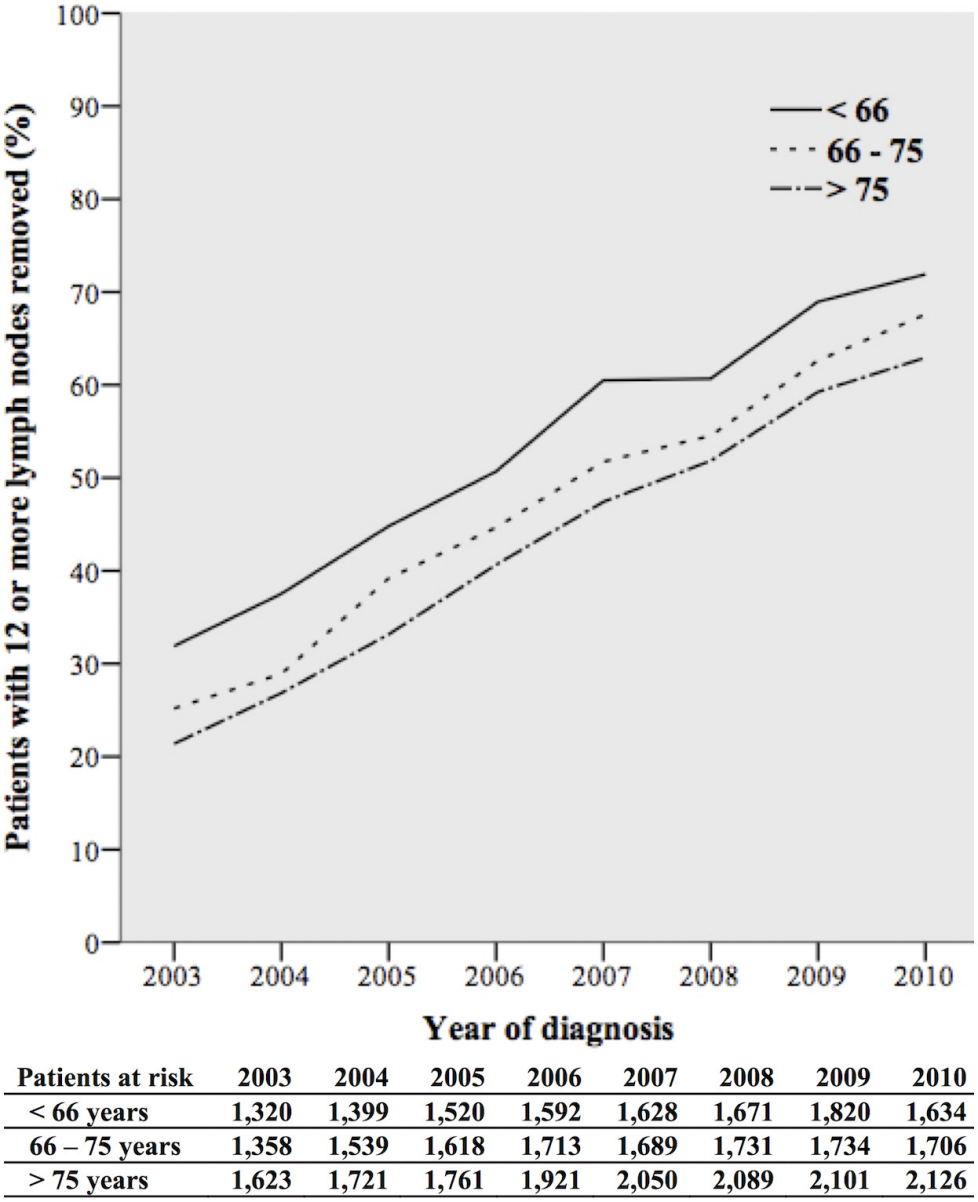
Patients with 12 or more lymph nodes removed per year of diagnosis, stratified by age groups.

**Fig 3:**
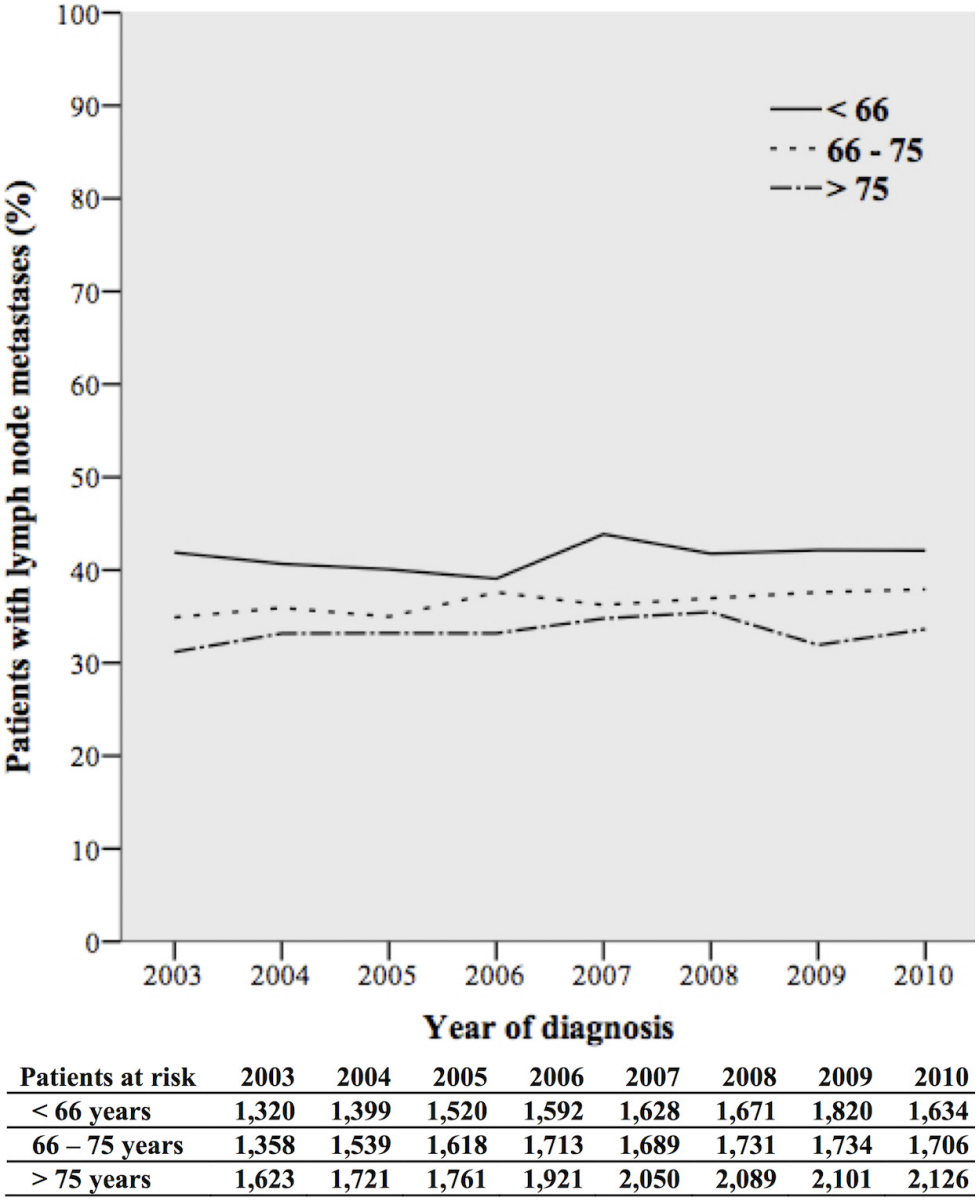
Patients diagnosed with lymph node metastases per year of diagnosis, stratified by age groups.

From 2003 through 2010 the removal of 12 or more lymph nodes increased from 25.8% to 67.1%. The percentage increased from 31.9% to 71.9% for patients younger than 66 years, from 25.2% to 67.6% in patients aged 66 to 75 years, and from 21.4% to 62.9% in patients older than 75 years (Fig 2). The percentage of patients diagnosed with lymph node metastases was rather stable from 2003 through 2010: 41.9% versus 42.1% in patients younger than 66 years, 34.9% to 37.9% in patients aged 66 to 75 years, and 31.2% to 33.6% in patients older than 75 years (Fig 3).

When analysing trends over time per age group, we found no changes in the location of the tumour or the stage of tumour. Even so, the percentage of patients per age group receiving chemotherapy remained stable over the study period. The percentage of patients per age group with 12 or more lymph nodes that received chemotherapy remained also stable over time.

Patients aged 66 to 75 years and patients older than 75 years, were less likely to have 12 or more lymph nodes removed, as compared to patients under the age of 66 (respectively OR: 0.718 (95% CI, 0.681–0.757) and OR 0.559 (95% CI, 0.531–0.589))(Table 2).

**Table 2:** Factors associated with the removal of 12 or more lymph nodes in patients with colon cancer (N = 41,074); multivariate logistic regression analysis.

Characteristics	OR (95% Cl)	*p*
Age		
< 66 years	1	< 0.001
66–75 years	0.718 (0.681–0.757)	< 0.001
> 75 years	0.559 (0.531–0.589)	< 0.001
Gender		
Men	1	
Female	1.057 (1.013–1.102)	0.011
Tumour location		
Right (+ transversum)	1	< 0.001
Left	0.560 (0.518–0.606)	< 0.001
Sigmoid	0.577 (0.551–0.604)	< 0.001
Morphology		
Adenocarcinomas	1	
Mucinous neoplasms	1.015 (0.958–1.076)	0.615
Differentiation grade		
Well differentiated	1	0.006
Moderately differentiated	1.082 (1.000–1.171)	0.051
Poorly differentiated	1.082 (0.986–1.188)	0.098
Unknown	0.950 (0.854–1.058)	0.351
Tumour invasion		
T1	1	< 0.001
T2	2.450 (2.189–2.744)	< 0.001
T3	4.002 (3.603–4.446)	< 0.001
T4	3.626 (3.221–4.082)	< 0.001
N status		
N0	1	< 0.001
N1	0.918 (0.873–0.965)	0.001
N2	1.770 (1.652–1.897)	< 0.001
Year of diagnosis		
2003	1	< 0.001
2004	1.279 (1.163–1.406)	< 0.001
2005	1.863 (1.699–2.042)	< 0.001
2006	2.501 (2.286–2.737)	< 0.001
2007	3.420 (3.127–3.740)	< 0.001
2008	3,832 (3.505–4.191)	< 0.001
2009	5.425 (4.958–5.936)	< 0.001
2010	6.661 (6.076–7.302)	< 0.001

### Overall and relative survival

**Fig 4:**
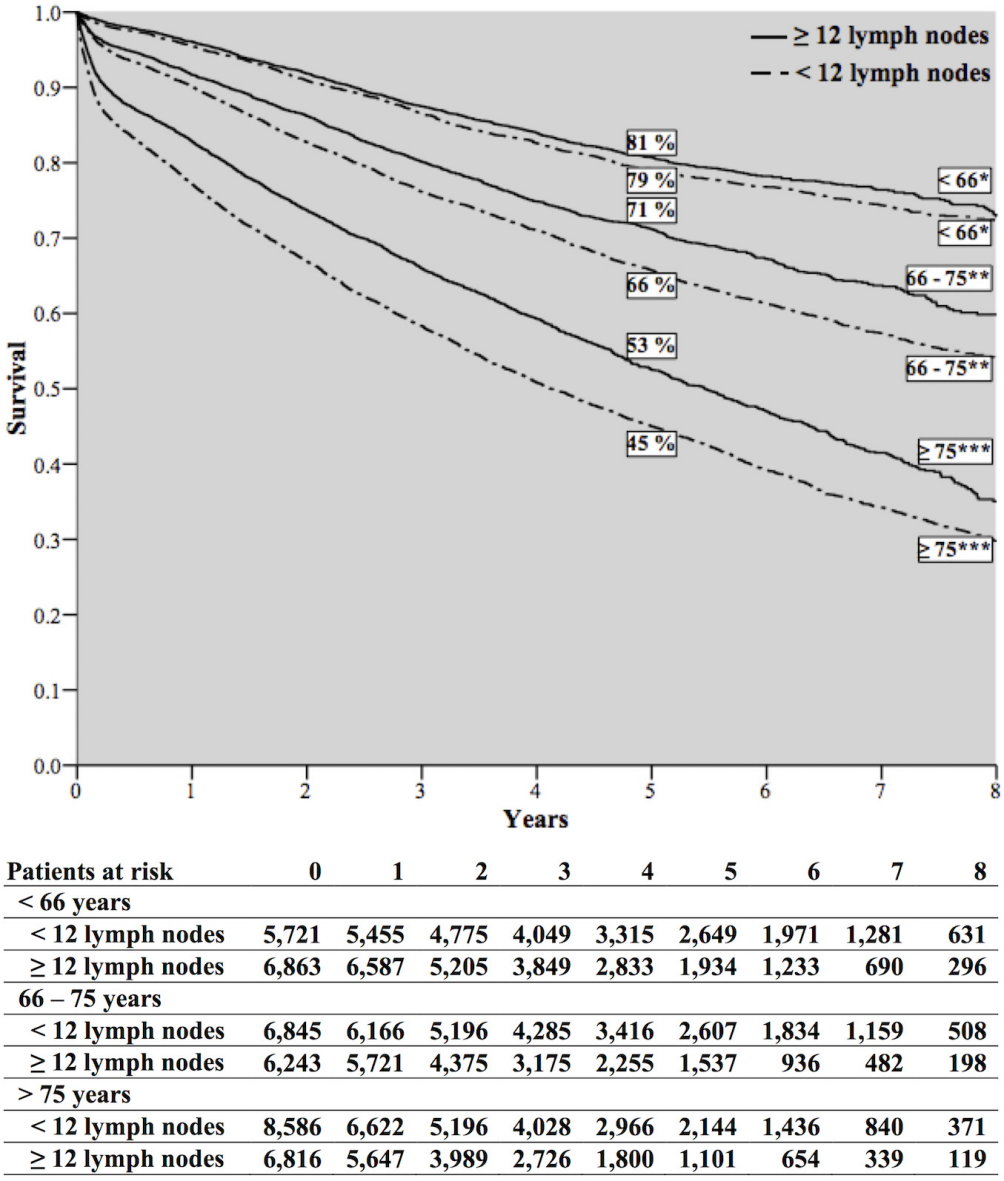
Survival in patients in all age groups with colon cancer stratified by the number of removed lymph nodes. *Log-rank: 3.573, *p* = 0.059; **Log-rank: 34.937, *p* < 0.001; ***Log-rank: 100.427, *p* < 0.001.

Overall survival decreased with age and if less than 12 lymph nodes were removed (Fig 4). Overall 5-year survival rates were 2% higher for patients younger than 66 years when 12 or more lymph nodes were removed: 81% versus 79%. In patients aged 66 to 75 years this difference was 5% (71% versus 66%) and in patients older than 75 years there was a 8% difference (53% versus 45%).

After adjustment for gender, tumour location, tumour morphology, differentiation grade, tumour invasion, the presence of lymph node metastases, year of diagnosis and adjuvant chemotherapy, it was found that for patients in which 12 or more lymph nodes were removed compared to those with less than 12, there was a statistically significant higher overall survival in all three age groups, (< 66: HR: 0.858 (95% CI, 0.789–0.933); 66–75: HR: 0.763 (95% CI, 0.714–0.814); > 75: HR: 0.734 (95% CI, 0.700–0.771)) (Table 3).

**Table 3:** The impact of the number of removed lymph nodes on overall and relative survival in all age groups (N = 41,074)^a^.

Characteristic	Overall survival	Relative survival
	HR (95%CI)^b^	RER (95% CI)^b^
< 66 years		
< 12 lymph nodes	1	1
≥ 12 lymph nodes	0.858 (0.789–0.933)	0.783 (0.708–0.865)
66–75 years		
< 12 lymph nodes	1	1
≥ 12 lymph nodes	0.763 (0.714–0.814)	0.672 (0.611–0.739)
> 75 years		
< 12 lymph nodes	1	1
≥ 12 lymph nodes	0.734 (0.703–0.774)	0.621 (0.567–0.681)

^a^Adjusted for gender, tumour location, tumour morphology, differentiation grade, tumour invasion, the presence of lymph node metastases, year of diagnosis and adjuvant chemotherapy.

^b^*p* < 0.001

After adjustment for the same patient and tumour characteristics and adjuvant chemotherapy, it was found that for patients in which 12 or more lymph nodes were removed compared to those with less than 12, there was a statistically significantly higher relative survival in all age groups (< 66: RER: 0.783 (95% CI, 0.708–0.865); 66–75: RER: 0.672 (95% CI, 0.611–0.739); > 75: RER: 0.621 (95% CI, 0.567–0.681)) (Table 3).

The benefit in survival in patients with 12 or more lymph nodes was present in both patients with a right-sided tumour and in patients with a tumour originated from the sigmoid (data not shown).

## Discussion

In this large population-based study of patients operated on colon cancer from 2003 through 2010, we found that over a time period of 8 years there was an increase of about 40% in patients with 12 or more lymph nodes removed, whereas the percentage of patients with lymph nodes metastases remained stable over this time period. After adjustment for patient and tumour characteristics and adjuvant chemotherapy, it was found that for patients in which 12 or more lymph nodes were removed, there was a statistically significant higher overall and relative survival in all age groups. This effect was stronger in the elderly.

The National Comprehensive Cancer Network, American Joint Committee on Cancer, and American College of Pathologist recommend the removal of at least 12 lymph nodes for adequate staging in colon cancer [7,16]. Because there is no clear cut-off point in the literature, the Dutch guidelines state that the removal of 10 or more lymph nodes is sufficient for adequate staging [17]. However, they also state that all the lymph nodes in a specimen should be evaluated. The initial rational is that the removal of more lymph nodes will improve accurate staging, so that more patients can benefit from adjuvant therapy [11,18]. Though the removal of less than 12 lymph nodes is not uncommon [13,19,20]. In accordance with our results, multiple studies show that there is an increase over time in the number of lymph nodes removed, whereas the number of patients diagnosed with lymph nodal metastases remains the same [21–24]. Currently the percentage of patients with 10 or more lymph nodes removed and 12 or more lymph nodes removed in the Netherlands is approximately 83% and 73% respectively, and thereby stable over the last years. Although there is no significant association between a higher number of removed lymph nodes and the detection of lymph node metastases, a higher number of removed lymph nodes is associated with improved relative survival [21]. An improvement in both overall and disease specific survival when more lymph nodes are removed was also reported in a systematic review from Chang et al [25]. These results suggest a more accurate staging is not the primary explanation for improved survival in patients with more lymph nodes removed.

In general an increased awareness amongst surgeons and pathologists resulted in a more diligence approach in the removal of more lymph nodes in colon cancer. Previous studies showed a large variation in adequate lymph node removal and evaluation between hospitals, suggesting differences in efforts of the surgeons and pathologists [13, 26]. They found that academic hospitals and pathology laboratories report a higher number of removed lymph nodes. Increased awareness as a result of feedback to surgeons and pathologists in multidisciplinary working groups may have contributed in the increase in number of removed lymph nodes per year of diagnosis [27]. At last, differences in the number of removed lymph nodes could be explained by differences in pathology reports between pathology laboratories [26,28].

Nevertheless, the increase in the number of removed lymph nodes corresponds with the improvement in survival in the past decades [29]. The removal of more lymph nodes is also important for other prognostic variables like the ratio between number of removal lymph nodes and the number of lymph node metastasis (lymph node ratio). Recently several studies demonstrated their important prognostic role in the prediction of survival in colon cancer. However the role of age is still underexposed [30,31].

In contrast to other studies, we focused on the role of age on the removal of lymph nodes and the improvement in survival in the past decade. We found that in all age groups there is a significant improvement in both overall and relative survival when 12 or more lymph nodes were removed compared to when less than 12 lymph nodes were removed. This effect was also observed in two other large studies [32,33]. Although both studies reported an overall survival benefit in elderly patients when 12 or more lymph nodes were removed, these results were not adjusted for confounding patient and tumour characteristics and adjuvant chemotherapy. As such, bias by indication could not be excluded in these two studies. Even so, these studies did not present data on relative survival.

The significant improvement in survival when 12 or more lymph nodes were removed was strongest in the elderly patients, both in the survival analyses and the adjusted analyses. An interesting question is how to explain the larger overall and relative survival benefit we found in patients over 75 years of age. One explanation is that patients over 75 years of age form a large and heterogeneous group varying from robust, physically active and mentally intact individuals, to those who are cognitively impaired and suffer from multiple chronic morbidities [34]. It might be that vital elderly patients undergo a more extensive resection that results in the removal of more lymph nodes. As we have no information on comorbidity and functional status of the patients included in this database, we could not adjust for these patient characteristics. Another explanation is the postulation that a more extensive removal of lymph nodes results in the removal of micrometastases [35]. Lymph nodes with the presence of micrometastases (0.2–2mm) are considered to be node negative according to the Dutch guidelines [36]. However, the presence of micrometastases has recently been associated with local recurrence and decrease in survival [37,38]. Therefore a more extensive resection might improve survival.

It is suggested that a more robust immune reaction could lead to improved survival [39,40]. The number of lymph nodes decreases with age, partially due to involution of lymph nodes [13,41]. However, a less pronounced anti tumour response is also observed in elderly patients [42,43]. Patients who mount a stronger immune response to their cancers may have larger and hence easier detectable lymph nodes in their resected mesentery. Therefore the survival benefit we found might be attributed to the patients’ health and vitality.

The elderly tend to have more right-sided tumours [44]. Our results confirm this. Right-sided tumours are associated with a worse prognosis when compared to left-sided tumours [45]. Although the elderly tend to have a prognostic worse tumour, the removal of more lymph nodes leads to an increase in survival in the elderly. During our study period there were no changes in location of the tumour.

Because bowel screening programs were only introduced in 2013, and during our study period there were only a few trials regarding bowel screening, these programs hardly influenced our results. Remarkably, despite the absence of bowel screening programs, our database contained a higher proportion of stage II cancers compared to stage III. Which was also reported by another population-based study in the Netherlands [46].

The percentage of patients per age group receiving chemotherapy during our study period remained stable. Also when dividing these patients within these age groups in patients with less than 12 and 12 or more lymph nodes, the percentage of patients that received chemotherapy remained stable over time for both categories. Therefore the influence of chemotherapy on the improvement in survival in patients with 12 or more lymph nodes is limited.

Although our study provides more insight into the relationship between age, number of removed lymph nodes and survival in a population-based setting, we acknowledge several data related limitations. Data were derived from a cancer registry, in which no data were available on comorbidity and functional status of the patient. No data were available on laparoscopic versus open and elective versus emergency procedures. Also no data were available on type, duration or completion of chemotherapy. Finally, no data were available on the presence of micrometastases in node negative cancers and on microsatellite (in) stability of a tumour. Recently microsatellite instability is related to high lymph node retrieval in colon cancer and is present in approximately 15% of all colon tumours [47]. All these unmeasured confounders might have biased our findings.

## Conclusion

In colon cancer, the removal of lymph nodes increased over an eight years time period, whereas the percentage of patients with lymph nodes metastases remained stable over this time period. The removal of 12 or more lymph nodes leads to an improvement in both overall and relative survival in all patients. This effect was independent from patient and tumour characteristics and adjuvant chemotherapy and was stronger in elderly patients. The biology behind this association is multifactorial and needs further clarification. The survival benefit may be the consequence of the performance of more extensive surgery on relatively healthy and vital elderly patients. However, also tumour and patients biology might play an important role.
